# Porcine sialoadhesin suppresses type I interferon production to support porcine reproductive and respiratory syndrome virus infection

**DOI:** 10.1186/s13567-020-00743-7

**Published:** 2020-02-24

**Authors:** Yingqi Liu, Rui Li, Songlin Qiao, Xin-xin Chen, Ruiguang Deng, Gaiping Zhang

**Affiliations:** 1 Key Laboratory of Animal Immunology of the Ministry of Agriculture, Henan Provincial Key Laboratory of Animal Immunology, Henan Academy of Agricultural Sciences, Zhengzhou, Henan 450002 China; 2 College of Animal Science and Veterinary Medicine, Henan Agricultural University, Zhengzhou, Henan 450002 China

## Abstract

Porcine reproductive and respiratory syndrome virus (PRRSV) is a significant threat to the global swine industry. Porcine sialoadhesin (poSn) has been previously shown to mediate PRRSV attachment and internalization. In the current study, we report its unidentified role in antagonism of type I interferon (IFN) production during PRRSV infection. We determined that poSn facilitated PRRSV infection via inhibition of type I IFN transcription. Mechanistically, poSn interacted with a 12 kDa DNAX-activation protein (DAP12), which was dependent on residues 51–57 within DAP12 transmembrane domain (TMD). PRRSV exploited the poSn-DAP12 pathway to attenuate activation of nuclear factor-kappa B (NF-κB). More importantly, the poSn-DAP12 pathway was involved in inhibiting poly (I:C)-triggered IFN production. All these results reveal a novel role of poSn in suppressing host antiviral responses, which deepens our understanding of PRRSV pathogenesis.

## Introduction

Porcine reproductive and respiratory syndrome (PRRS) has been causing significant economic losses to the global swine industry [1]. Its clinical signs are respiratory distress and reproductive failure [2]. PRRS virus (PRRSV), as the causative agent, belongs to the *Porarterivirus* genus*, Arteriviridae* family in the order *Nidovirales* [3]. It is a single-stranded positive RNA virus with a genome of 14.9 to 15.5 kb in length. All PRRSV isolates are classified into PRRSV-1 and PRRSV-2, and PRRSV-2 strains are predominantly prevalent in China [4].

Porcine sialoadhesin (poSn) was first identified to be involved in PRRSV entry [5]. Subsequent research indicated that poSn is responsible for virus attachment and internalization, which is dependent on the sialic acid-binding activity of its N-terminal immunoglobulin (Ig)-like domain [6–8]. Non-permissive cells with co-expression of recombinant poSn and CD163 produce much more viral progenies than that expressing CD163 alone [9]. However, a recent report demonstrated that poSn knockout pigs are still susceptible to PRRSV [10]. These studies suggested that poSn might play certain unappreciated roles instead of an indispensable receptor during PRRSV infection.

poSn is a member of the sialic acid-binding Ig-like lectin (Siglec) family, namely Siglec-1 [11, 12]. Increasing evidence has shown that Siglecs modulate type I interferon (IFN) responses during viral infections. For example, Siglec-G is reported to be induced and exploited by RNA viruses to inhibit retinoic acid-inducible gene-I (RIG-I)-mediated type I IFN production [13]. Siglec-H is demonstrated to negatively regulate IFN-α production in response to murine cytomegalovirus infection in vitro and in vivo [14]. Murine Siglec-1 has been recently shown to inhibit IFN responses through impairing tank binding kinase 1 (TBK1)-interferon regulatory factor (IRF)-3 pathway during vesicular stomatitis virus (VSV) infection [15]. As a Siglec, whether poSn plays an immunosuppressive role during PRRSV infection has not been elucidated.

In this work, we unraveled that PRRSV utilized poSn to repress type I IFN production in favor of its infection. poSn associated with DNAX-activation protein of 12 kDa (DAP12) to attenuate PRRSV-triggered nuclear factor-kappa B (NF-κB) activation. More importantly, poSn-DAP12 pathway negatively modulated transcription of type I IFNs in response to poly (I:C), suggesting that the pathway might be involved in maintaining homeostasis by avoiding excessive immune responses.

## Materials and methods

### Cells and virus

Pulmonary alveolar macrophages (PAMs) were obtained from lung lavage samples of 4-week-old pigs. CRL-2843-CD163 (the continuous PAM cell line stably expressing porcine CD163), MARC-145 (the derivative from African green monkey kidney cell line MA-104) and HEK-293T (human embryonic kidney 293 cell line stably expressing SV40 large T antigen) cells were used in our studies. HEK-293T and MARC-145 cells were maintained in Dulbecco modified Eagle medium (DMEM, Solarbio life sciences, Beijing, China) supplemented with 10% heat-inactivated fetal bovine serum (FBS, Gibco, Logan, UT, USA) and penicillin–streptomycin mixtures (Solarbio life sciences). PAMs and CRL-2843-CD163 cells were cultured in Roswell Park Memorial Institute-1640 medium (RPMI-1640, Solarbio life sciences) supplemented with 10% FBS and antibiotics.

A typical PRRSV-2 strain BJ-4 (GenBank accession no. AF331831) was a gift from Professor Hanchun Yang of China Agricultural University. rBJ4-EGFP was constructed by inserting enhanced green fluorescent protein (EGFP) between open reading frame (ORF) 1b and ORF2a of PRRSV strain BJ-4 in our laboratory. These viruses used in our study were propagated in MARC-145 cells in DMEM with 3% FBS, and the virus titers were measured by 50% tissue culture infective dose (TCID_50_) assay in MARC-145 cells [16].

### Antibodies and reagents

Antibodies: Mouse anti-poSn monoclonal antibody (mAb, clone 3B11/11) was purchased from LifeSpan BioSciences (LSBio, Seattle, WA, USA). Mouse anti-DAP12 mAb was from Santa Cruz Biotechnology (Santa Cruz, CA, USA). Rabbit anti-IRF-3, phospho-IRF-3 (Ser386), NF-κB p65 (D14E12), phospho-NF-κB p65 (Ser536) (93H1), myc-tag (71D10), Flag (DYKDDDDK)-tag (D6W5B), Glyceraldehyde-3-phosphate dehydrogenase (GAPDH) (D16H11) and DAP12 mAbs, as well as mouse anti-nuclear factor of kappa light polypeptide gene enhancer in B-cells inhibitor-alpha (IκBα) (L35A5), myc-Tag (9B11), Flag (DYKDDDDK) tag (9A3) and β-actin (8H10D10) mAbs were all purchased from Cell Signaling Technology (CST, Boston, MA, USA).

Reagents: In-Fusion HD Cloning Kit was purchased from TaKaRa (Dalian, Liaoning, China). Poly (I:C) and bovine serum albumin (BSA) were purchased from Sigma-Aldrich (St. Louis, MO, USA). pGL3-basic vector and pRL-TK control vector were from Promega (Madison, WI, USA). pcDNA3.1-myc-hisA was purchased from Invitrogen (Carlsbad, CA, USA) and p3 × Flag-CMV-7.1 was from Sigma-Aldrich [17].

### Quantitative real-time PCR (RT-qPCR)

Total RNAs were extracted using TRIzol reagents (Thermo Fisher Scientific, Hanover Park, IL, USA) from the indicated cells and reverse-transcribed into cDNA by PrimeScript™ RT Reagent Kit with gDNA Eraser (TaKaRa) according to the manufacturer’s instructions. RT-qPCR was performed using the Universal SYBR Green Master (Roche, Basel, Basel-Stadt, Switzerland) on the 7500 Fast RT-PCR system (Applied Biosystems, Foster City, CA, USA). PCR was conducted with 1 μL of cDNA with primers specific for PRRSV ORF7, poSn, DAP12, IFN-α and IFN-β (Table 1). GAPDH was set as the endogenous control. Data analysis of relative gene expression was applied to the 2^−ΔΔCt^ method [18].

**Table 1:** **Primers for RT-qPCR and plasmid construction**

Primers	Sequence (5′–3′)
pig GAPDH-Forward	CCTTCCGTGTCCCTACTGCCAAC
pig GAPDH-Reverse	GACGCCTGCTTCACCACCTTCT
pig IFN-β-Forward	TGCAACCACCACAATTCC
pig IFN-β-Reverse	CTGAGAATGCCGAAGATCTG
pig IFN-α-Forward	GCCTCCTGCACCAGTTCTACA
pig IFN-α-Reverse	TGCATGACACAGGCTTCCA
poSn-Forward	CGTGTTGTGGCCTCTTCTCT
poSn-Reverse	CACGTTGCAAGTCAGGTTGG
pig DAP12-Forward	ACCCGGAAACAACACATCGC
pig DAP12-Reverse	TACTGCCTCTGTGTGTTGAGG
PRRSV-ORF7-Forward	AAACCAGTCCAGAGGCAAGG
PRRSV-ORF7-Reverse	GCAAACTAAACTCCACAGTGTAA
poSn-infusion-Forward	CTGGCTAGTTAAGCTATGGACTTCCTGCTCCTGCT
poSn-infusion-Reverse	GCCCTCTAGACTCGAGGACTGTGCTTTTCACAGA
poSn-ECD-infusion-Reverse	GCCCTCTAGACTCGAGGTGCTGGAACAGATGCA
poSn-TCD-Forward	CCAAGCTTGCCACCATGCTTCTCTGGTTCCT
poSn-TCD-Reverse	CCGCTCGAGGACTGTGCTTTTCACAGACTG
DAP12-Forward	CCCAAGCTTGCCACCATGGGAAGACTGGGGCCAT
DAP12-XhoI-Reverse	CCGCTCGAGTCACTTGTAGTACTGCCGCTGGGTA
DAP12-ΔICD-Forward	CGGAATTCTCAGAGAGAATGCAGCTGCT
DAP12-ΔICD-Reverse	GCTCTAGATCACACAGCCAGGGCGAT
DAP12-ΔECD-Forward	CGGAATTCTGGCATCCTGGCG
DAP12-ΔECD-Reverse	GCTCTAGATTTGTAATACTGCCTCTGTGTGTTG
DAP12(D50A)-Forward	GGGGATCTGGTGCTGGCCCTCCTCATCGCCCTG
DAP12(D50A)-Reverse	CAGGGCGATGAGGAGGGCCAGCACCAGATCCCC
DAP12-ΔTM1-Forward	GCTGCTCCGCCGTGAGCCCCCTGGGGGATCTGGTGCTGAC
DAP12-ΔTM1-Reverse	GTCAGCACCAGATCCCCCAGGGGGCTCACGGCGGAGCAGC
DAP12-ΔTM2-Forward	GCATCCTGGCGGGGATCGTGCTCCTCATCGCCCTGGCTGT
DAP12-ΔTM2-Reverse	ACAGCCAGGGCGATGAGGAGCACGATCCCCGCCAGGATGC
DAP12-ΔTM3-Forward	TGGGGGATCTGGTGCTGACCTACTCCCTGGGTCGGCTGGT
DAP12-ΔTM3-Reverse	ACCAGCCGACCCAGGGAGTAGGTCAGCACCAGATCCCCCA

### Immunoblotting (IB)

The indicated cells were lysed with radio immunoprecipitation assay (RIPA) lysis buffer (Beyotime Biotechnology, Shanghai, China) supplemented with protease inhibitor cocktail (Roche). After boiling, the indicated samples were subjected to sodium dodecyl sulfate–polyacrylamide gel electrophoresis (SDS-PAGE) followed by transferring onto polyvinylidene fluoride (PVDF) membranes (Merck Millipore, Burlington, MA, USA). The membranes were blocked with 5% skimmed-milk at room temperature (RT) for 2 h, and then incubated with the specific primary antibodies at RT for 2 h. After extensive washing with phosphate-buffered saline-Tween 20 (PBST), the membranes were incubated with the corresponding horse radish peroxidase (HRP)-conjugated secondary antibodies at RT for 1 h. An enhanced chemiluminescence (ECL) detection system was used to detect the indicated proteins (Solarbio life sciences).

### Flow cytometry (FCM) analysis

After trypsin treatment, the transfected PAMs were collected and washed with PBS twice. The cells were centrifuged (200 × *g*) at 4 °C for 5 min and subsequently resuspended in 2% BSA-PBST buffer at 4 °C for 15 min. After centrifugation, the cells were incubated with the commercial anti-poSn mAb in 2% BSA-PBST buffer at 4 °C for 1 h. After washing with PBST 3 times, the cells were then incubated with Dylight 649 (red) conjugated goat anti-rabbit IgG (H+L) secondary antibody (Thermo Fisher Scientific) in 2% BSA-PBST buffer at 4 °C for 30 min. After washing, the cells were resuspended in 0.5% paraformaldehyde (PFA) in BSA-PBST buffer. Based on the acquisition of 2.0 × 10^4^ cells, the data were analyzed using CytoFLEX (Beckman Coulter, Brea, CA, USA).

### Knockdown assays

Small interfering RNAs (siRNAs) targeting poSn or DAP12 (Table 2) were designed and synthesized by GenePharma (Shanghai, China). Transfection of siRNA was conducted in PAMs with Lipofectamine RNAiMAX Reagent (Thermo Fisher Scientific) for the indicated time periods (35 h or 47 h). The knockdown efficiencies were determined by RT-qPCR or FCM analyses.

**Table 2:** **siRNA**

siRNA names	5′–3′ (sense)	5′–3′ (anti-sense)
sipoSn-332#	GCUCCUAUAACUUCCGCUUTT	AAGCGGAAGUUAUAGGAGCTT
sipoSn-1983#	CCGCAUGAAGGUCACCAAATT	UUUGGUGACCUUCAUGCGGTT
siDAP12-433#	GGAUACGGAUCCACAGAGUTT	ACUCUGUGGAUCCGUAUCCTT
siRNA-NC	UUCUCCGAACGUGUCACGUTT	ACGUGACACGUUCGGAGAATT

### Plasmid construction and overexpression

All target genes were cloned from PAM cDNA. Complete poSn, poSn-extracellular domain (ECD, residues 1–1642, the numbering is according to UniProt entry A7LCJ3) and poSn-helical transmembrane plus cytoplasmic domain (TCD, residues 1643–1730) were cloned into pcDNA3.1-mychisA. DAP12, DAP12-Δ intracellular domain (ICD, residues 1–57, the numbering is according to UniProt entry Q9TU45), DAP12-ΔECD (residues 37–108), DAP12-ΔTM1 (the absence of residues 37–43), -ΔTM2 (the absence of residues 44–50) and -ΔTM3 (residues 51–57) were inserted into p3×Flag-CMV-7.1, respectively. Overexpression assays were performed with transfection of the indicated plasmids by Lipofectamine^®^ LTX with Plus™ Reagent according to Thermofisher’s instructions in CRL-2843-CD163, or HEK-293T cells. The primers were listed in Table 1. All constructs were verified by Shanghai Sangon Biotechnology (Shanghai, China).

### Dual-luciferase assays

Luciferase assays were conducted by Dual-Luciferase^®^ Reporter Assay System according to Promega’s instructions. In brief, CRL-2843-CD163 cells were transfected with 1 μg pig IFN-β-promoter [19] and 100 ng pRL-TK renilla luciferase reporter plasmid as an internal control, and then transfected with 350 ng 3×Flag-DAP12 and 650 ng poSn-myc-his. pRIG-I [19] was used to stimulate the activity of pig IFN-β promoter. The transfected cells were lysed in passive lysis buffer and subjected to luciferase activity measurement.

### Indirect immunofluorescence assay (IFA)

PRRSV-infected PAMs were fixed with 4% PFA buffer (Solarbio life sciences) at RT for 15 min followed by membrane permeabilization with 0.2% Tween-20. The cells were then incubated with mouse anti-poSn mAb and rabbit anti-DAP12 mAb in 2% BSA-PBST buffer at 4 °C overnight. After washing with PBST, the cells were incubated with DyLight 405 (blue) conjugated goat anti-mouse IgG (H+L) secondary antibody and Dylight 649 (red) conjugated goat anti-rabbit IgG (H+L) secondary antibody (Thermo Fisher Scientific) at 4 °C for 1 h, respectively. After washing, the cells with SlowFade^®^ Gold buffer (Life Technologies, Carlsbad, CA, USA) were visualized by a laser scanning confocal microscope (LSM 800, Carl Zeiss AG, Oberkochen, Germany).

### Co-immunoprecipitation (Co-IP)

Transfected HEK-293T cells were lysed in IP lysis buffer (Beyotime Biotechnology) at 4 °C for 30 min. After centrifugation at 12 000 *g* at 4 °C for 15 min, whole cell lysates (WCLs) were harvested to mix with anti-myc or anti-Flag mAbs (CST), and then incubated with Protein A/G beads (GE Healthcare, Pittsburgh, PA, USA) at the rotator at 4 °C for 3 h or overnight. After extensive washing with Tris-buffered saline with 0.5% Tween-20 (TBST), the beads were vortexed with elution buffer (0.05 M glycine–HCl buffer pH 2.2) thoroughly. The eluted proteins were subjected to IB.

### Poly (I: C) stimulation

Poly (I:C), the synthetic analog of double-stranded RNA (dsRNA), is experimentally used to trigger type I IFN production [20]. We transfected the various amounts (0.25, 2 or 2.5 μg/mL) of poly (I:C) into CRL-2843-CD163 cells or *poSn* (or *DAP12*) knockdown PAMs with Lipofectamine RNAiMAX Reagent for the indicated time points (0, 2, 4, 12 h or 0, 3, 6 h). The cells were then subjected to RT-qPCR to detect the transcription of poSn and IFN-α/β.

### Statistical analysis

All experiments were independently repeated at least 3 times and each experiment included at least three replicates. RT-qPCR data were analyzed using Student *t* test method with GraphPad Software (San Diego, CA, USA), and denoted as mean ± standard error of the mean (SEM). The asterisk (*) indicated for statistical significance: *p < 0.05, **p <0.01, ***p <0.001; ns: not significant.

## Results

### poSn facilitates PRRSV infection and inhibits PRRSV-induced *IFN-α/β* transcription

**Figure 1:**
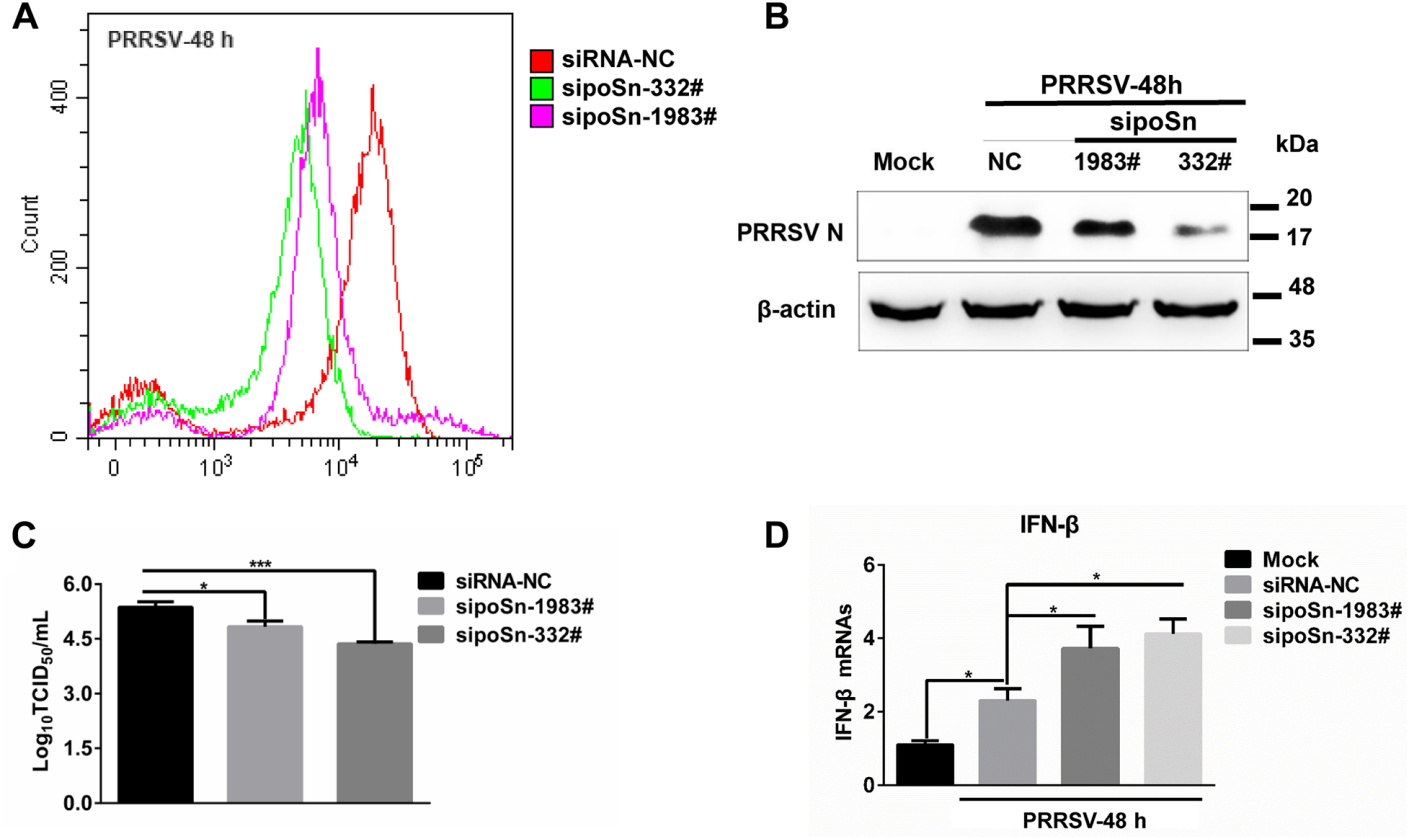
**poSn promotes PRRSV infection by inhibiting PRRSV-triggered *IFN***-***β*transcription.A**–**D** PAMs were pre-inoculated with PRRSV (MOI = 1) at 37 °C for 1 h. After washing with serum-free RPMI-1640, PAMs were transfected with sipoSn-332# or sipoSn-1983# for 47 h. *poSn* knockdown was determined by FCM **A**. IB was used to detect the abundance of PRRSV N protein **B**. PRRSV TCID_50_/mL of supernatants was measured, which was independently repeated three times **C**. *IFN*-*β* transcription was detected by RT-qPCR, which was independently repeated three times **D**. Data were denoted as mean ± SEM. Statistical analysis was applied to Student *t* test: *p < 0.05, ***p < 0.001. FCM images were representative from two independent experiments.

To verify the biological significance of poSn during PRRSV infection, we examined the effects of *poSn* knockdown on PRRSV infection and PRRSV-triggered type I IFN production. We pre-inoculated PRRSV into its primary in vivo target, PAMs [21], and then extensively washed the cells with serum-free medium followed by *poSn* knockdown assays. Two siRNAs (332# and 1983#) targeting poSn were synthesized and transfected into PAMs. The knockdown efficiencies were determined by FCM analysis (Figure 1A). First, we determined PRRSV replication by detecting expression of the viral nucleocapsid (N) protein. *poSn* knockdown decreased the abundance of PRRSV N protein (Figure 1B). Subsequently, we measured the viral titers from the supernatants of *poSn* knockdown or untreated PAMs. *poSn* knockdown suppressed PRRSV release as shown by TCID_50_ assay (Figure 1C). Furthermore, we checked PRRSV-induced transcription of type I IFN after *poSn* knockdown. *poSn* knockdown promoted the *IFN*-*β* transcription (Figure 1D), which suppressed PRRSV infection (Figures 1B and C). All these results suggest that poSn facilitates PRRSV infection by inhibiting the virus-triggered type I IFN production.

### poSn is determined to interact with DAP12 during PRRSV infection

**Figure 2:**
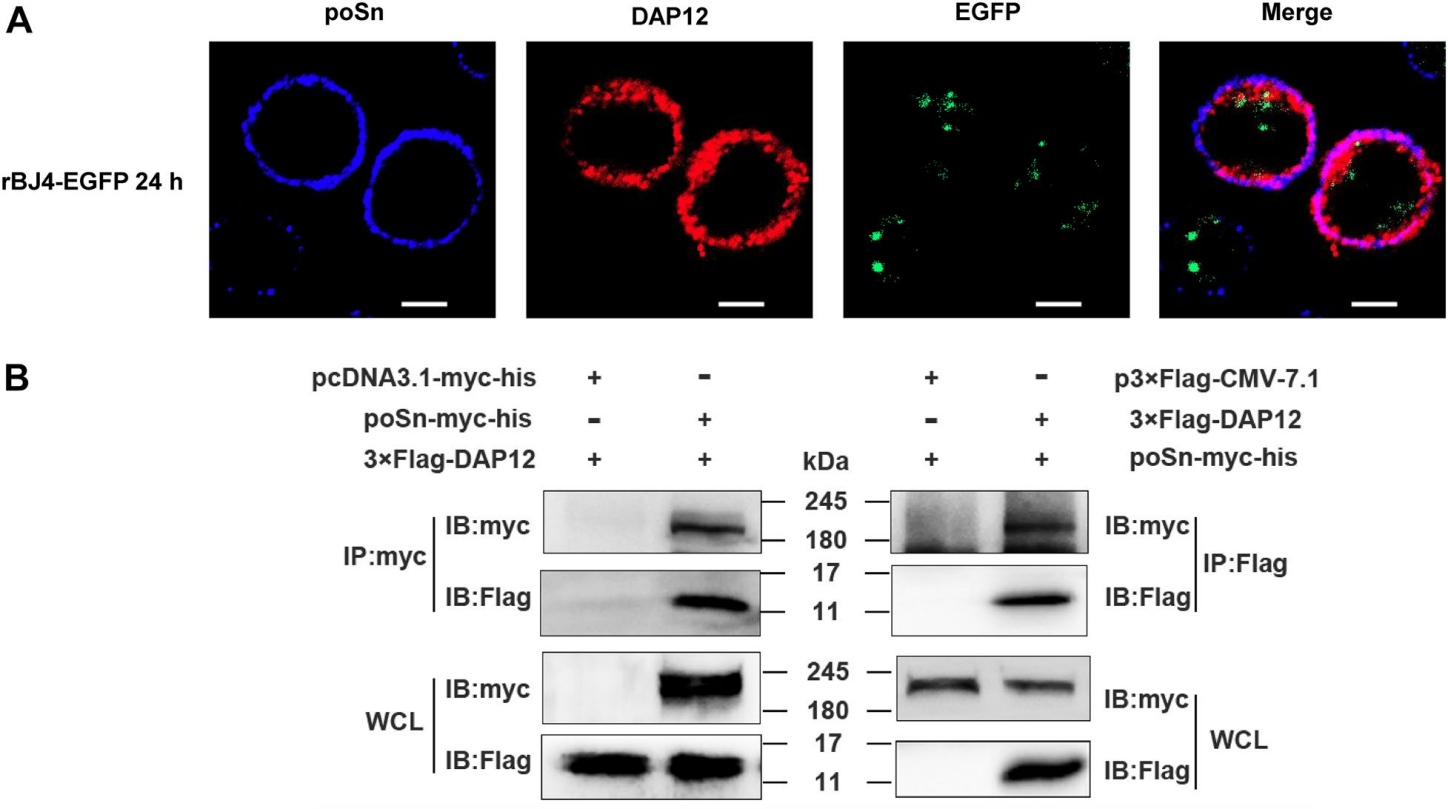
**poSn interacts with DAP12 in PRRSV-infected PAMs.A** PAMs were infected with rBJ4-EGFP (MOI = 1) for 24 h. Cells were fixed by 4% PFA for 15 min at RT and permeabilized with 0.2% Tween-20 for membrane protein staining. poSn was stained with DyLight 405 (blue) and DAP12 was stained with DyLight 649 (red). The co-localization of the two proteins was visualized by confocal microscopy. Scale bars, 10 μm. **B** HEK-293T cells were co-transfected with poSn-myc-his (10 μg) and 3×Flag-DAP12 (6 μg) for 48 h. WCLs were subjected to IP assays with anti-myc mAb or anti-Flag mAb. IB was performed to detect the indicated proteins. The confocal images were representative from two independent experiments, and IB panels were representative from three independent experiments.

Murine Siglec 1, the homolog of poSn, has been reported to inhibit type I IFN production by interacting with DAP12 during VSV infection [15]. To dissect the mechanism by which poSn played the IFN-suppressive role during PRRSV infection, we examined whether poSn interacted with DAP12 upon viral infection. We first observed that poSn co-localized with DAP12 by confocal microscopy in PRRSV-infected PAMs (Figure 2A). Subsequently, we immunoprecipitated DAP12 or poSn from the WCLs of HEK-293T cells co-transfected with poSn-myc-his and 3×Flag-DAP12. The Co-IP results confirmed the interaction between poSn and DAP12 (Figure 2B).

**Figure 3:**
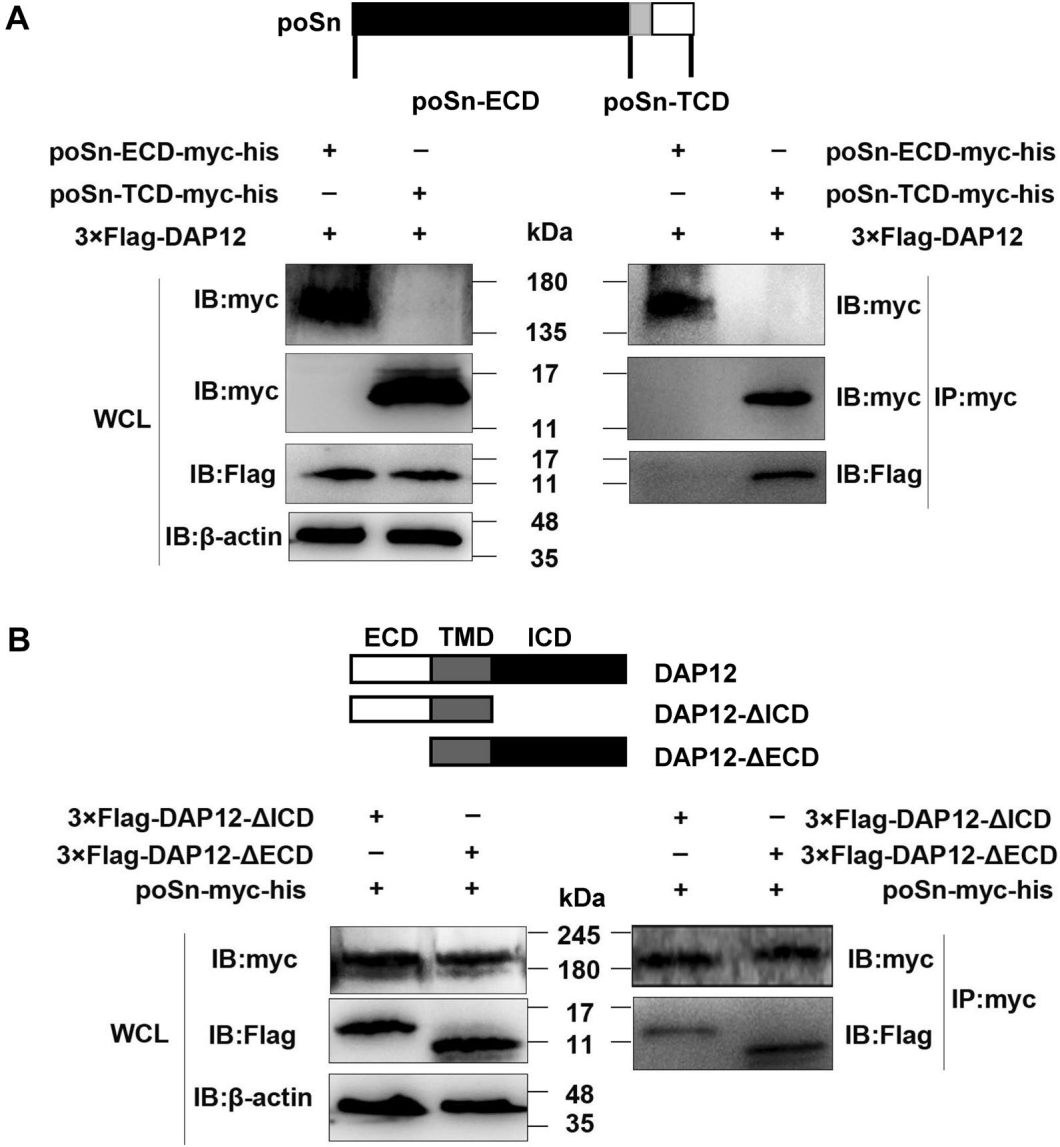
**poSn-TCD and DAP12 TMD are responsible for their interaction.A** HEK-293T cells were transfected with 3×Flag-DAP12 (6 μg) and poSn-TCD-myc-his (6 μg) or poSn-ECD-myc-his ( μg) for 48 h. WCLs were subjected to IP assays with anti-myc mAb at 4 °C for 3 h. IB was used to detect the indicated proteins. **B** IP assays with anti-myc mAb were performed using WCLs from HEK-293T cells co-transfected with poSn-myc-his (10 μg) and 6 μg 3×Flag-DAP12-ΔICD or 3×Flag-DAP12-ΔECD for 48 h. IB analysis was performed to examine the specific proteins. IB panels were representative from three independent experiments.

Next, we explored how poSn interacted with DAP12. We divided poSn into two fragments, the poSn-ECD (residues 1–1642) and poSn-TCD (residues 1643–1730), into pcDNA3.1-mychisA as poSn-ECD-myc-his and poSn-TCD-myc-his, respectively. We conducted IP assays with anti-myc mAb and WCLs from HEK-293T co-transfected with 3×Flag-DAP12 and poSn-ECD-myc-his or poSn-TCD-myc-his. IB analysis showed that poSn ECD was not required for its interaction with DAP12 (Figure 3A).

We constructed two DAP12 fragments: 3×Flag-DAP12-ΔICD (residues 1–57), DAP12 with deletion of ICD, and 3×Flag-DAP12-ΔECD (residues 37–108), DAP12 with deletion of ECD. WCLs were subjected to IP analysis from HEK-293T cells co-transfected with poSn-myc-his and 3×Flag-DAP12-ΔICD or 3×Flag-DAP12-ΔECD. We found that DAP12-ΔICD and DAP12-ΔECD both bound to poSn, suggesting that DAP12 transmembrane domain (TMD) was responsible for its interaction with poSn (Figure 3B).

**Figure 4:**
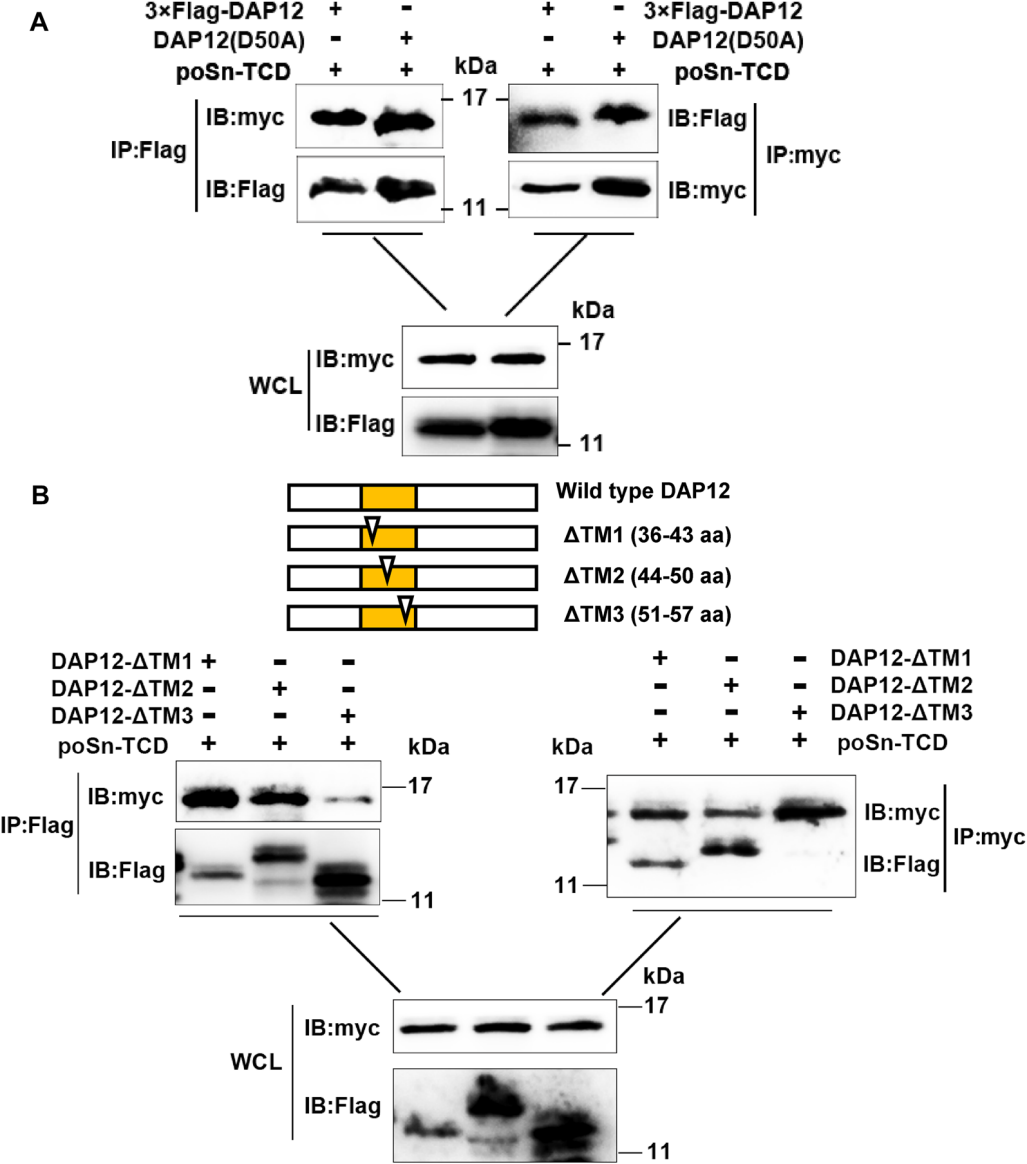
**Residues 51–57 are responsible for interaction between DAP12 and poSn.A** HEK-293T cells were co-transfected with 6 μg poSn-TCD-myc-his and 6 μg 3×Flag-DAP12 or DAP12-D50A for 48 h. Co-IP experiments were performed with anti-Flag mAb or anti-myc mAb. The indicated proteins were analyzed by IB from eluted proteins and WCLs. **B** HEK-293T cells were co-transfected with 6 μg poSn-TCD-myc-his and 6 μg 3×Flag-DAP12 or the indicated DAP12 mutants (DAP12-ΔTM1, ΔTM2 or ΔTM3) for 48 h. WCLs were incubated with Protein A/G beads bound to anti-Flag or anti-myc mAb at 4 °C overnight. IB analysis was conducted for detecting DAP12, DAP12 mutants or poSn-TCD. IB panels were representative from three independent experiments.

The negatively charged aspartic acid at position 50 (D50) of DAP12 is essential for its electrostatic interaction with certain immunoreceptors possessing positively charged residues within their TMDs [22, 23]. We sought to determine whether D50 was required for DAP12′s association with poSn. We mutated D50 to alanine (DAP12-D50A), and found that this mutation had no effects on the interaction (Figure 4A). We further deleted partial residues within DAP12 TMD and obtained three DAP12 mutants (DAP12-ΔTM1, -ΔTM2, and -ΔTM3) [17]. Using anti-Flag mAb to immunoprecipitate Flag-tagged DAP12 or its mutants, we demonstrated that residues 51–57 were indispensable for the interaction between DAP12 and poSn-TCD (Figure 4B). The IP assay with anti-myc mAb also confirmed the above result (Figure 4B). Collectively, these results revealed that, in PRRSV-infected cells, poSn interacted with DAP12 dependent on poSn TCD and DAP12 residues 51–57.

### poSn-DAP12 pathway mediates inhibition of type I IFN production during PRRSV infection

**Figure 5:**
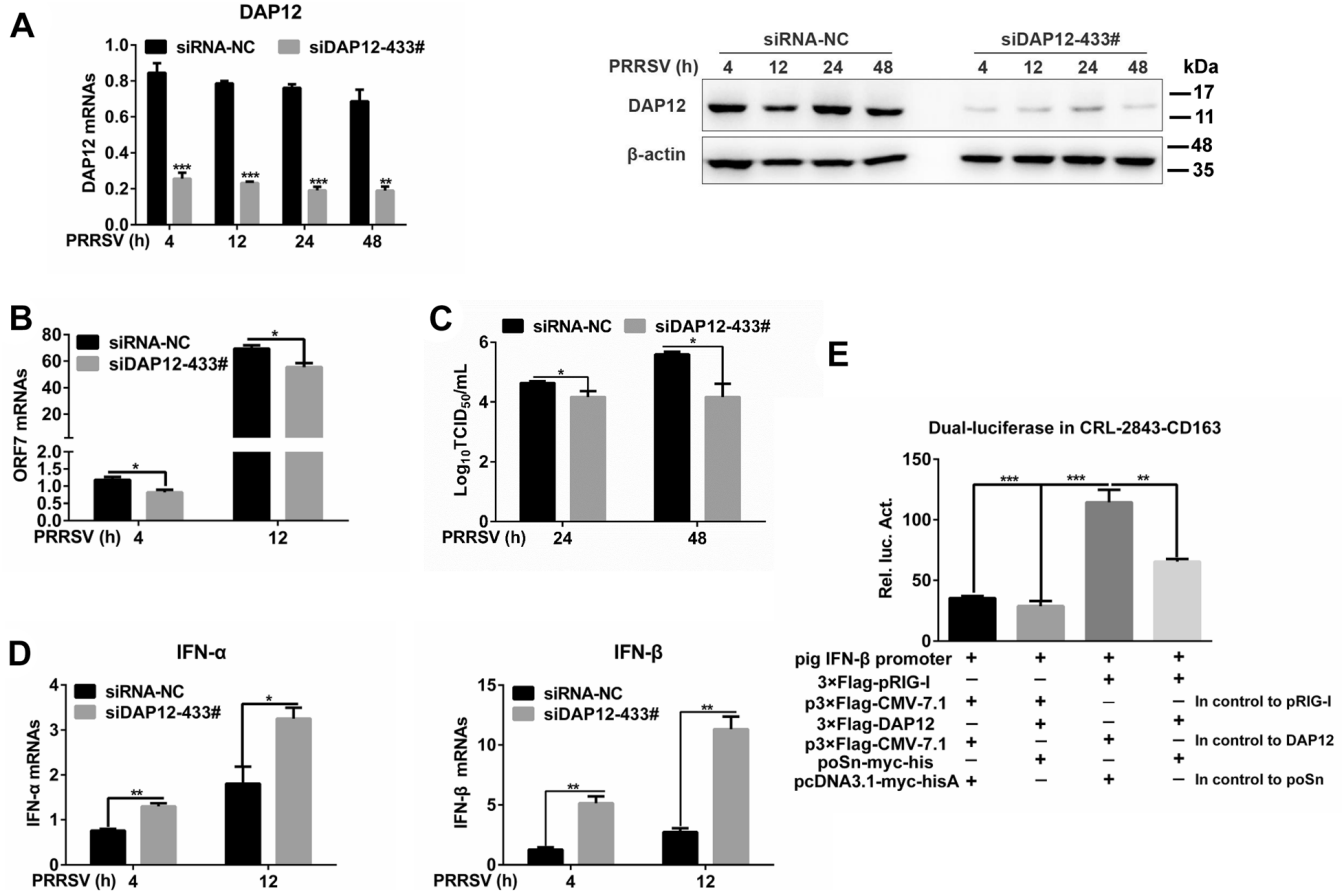
**poSn-DAP12 pathway facilitates PRRSV infection through suppressing type I IFN production.A**–**D** PAMs were transfected with siDAP12-433# for 36 h, and then infected with PRRSV (MOI = 1) for the indicated time periods (4, 12, 24, 48 h). *DAP12* knockdown was determined by RT-qPCR and IB **A**. PRRSV ORF7 was examined by RT-qPCR at 4 or 12 h post-infection **B**. TCID_50_ assay was performed to measure the viral titers at 24 or 48 h post-infection **C**. RT-qPCR was used to detect transcription of IFN-α/β at 4 or 12 h post-infection **D**, **E** Pig IFN-β promoter was activated by 3×Flag-pRIG-I overexpression, and the relative luciferase activity (Rel. luc. Act) of pig IFN-β promoter was measured in CRL-2843-CD163 cells transfected with poSn-myc-his and/or 3×Flag-DAP12 by dual-luciferase reporter assays. Data were indicated as mean ± SEM. Statistical significances were shown by Student *t* test: *p < 0.05, **p <0.01, ***p <0.001.

Since DAP12 was a binding partner of poSn during PRRSV infection, we investigated the effects of *DAP12* knockdown on PRRSV-triggered *IFN*-*α/β* transcription. As shown in Figures 5A and B, knockdown of DAP12 (siDAP12-433#) suppressed PRRSV replication as indicated by decreased PRRSV ORF7 mRNA levels. We further evaluated the viral release by TCID_50_ assay. *DAP12* knockdown decreased the viral titers from the supernatants of PRRSV-infected PAMs (Figure 5C). In contrast, *DAP12* knockdown increased the mRNA abundance of IFN-α/β in response to PRRSV (Figure 5D). These results were similar to that in *poSn* knockdown PAMs (Figure 1). Moreover, we conducted dual-luciferase assays in the continuous PAM cell line CRL-2843-CD163 that stably expresses porcine CD163 and is permissive to PRRSV [24]. Co-expression of poSn and DAP12 inhibited IFN-β promoter activation triggered by pig RIG-I (pRIG-I) overexpression (Figure 5E). Together, these results suggest that the poSn-DAP12 pathway is involved in restraining PRRSV-induced type I IFN production, which is beneficial for the viral infection.

### poSn-DAP12 pathway is involved in suppression of NF-κB activation in response to PRRSV

**Figure 6:**
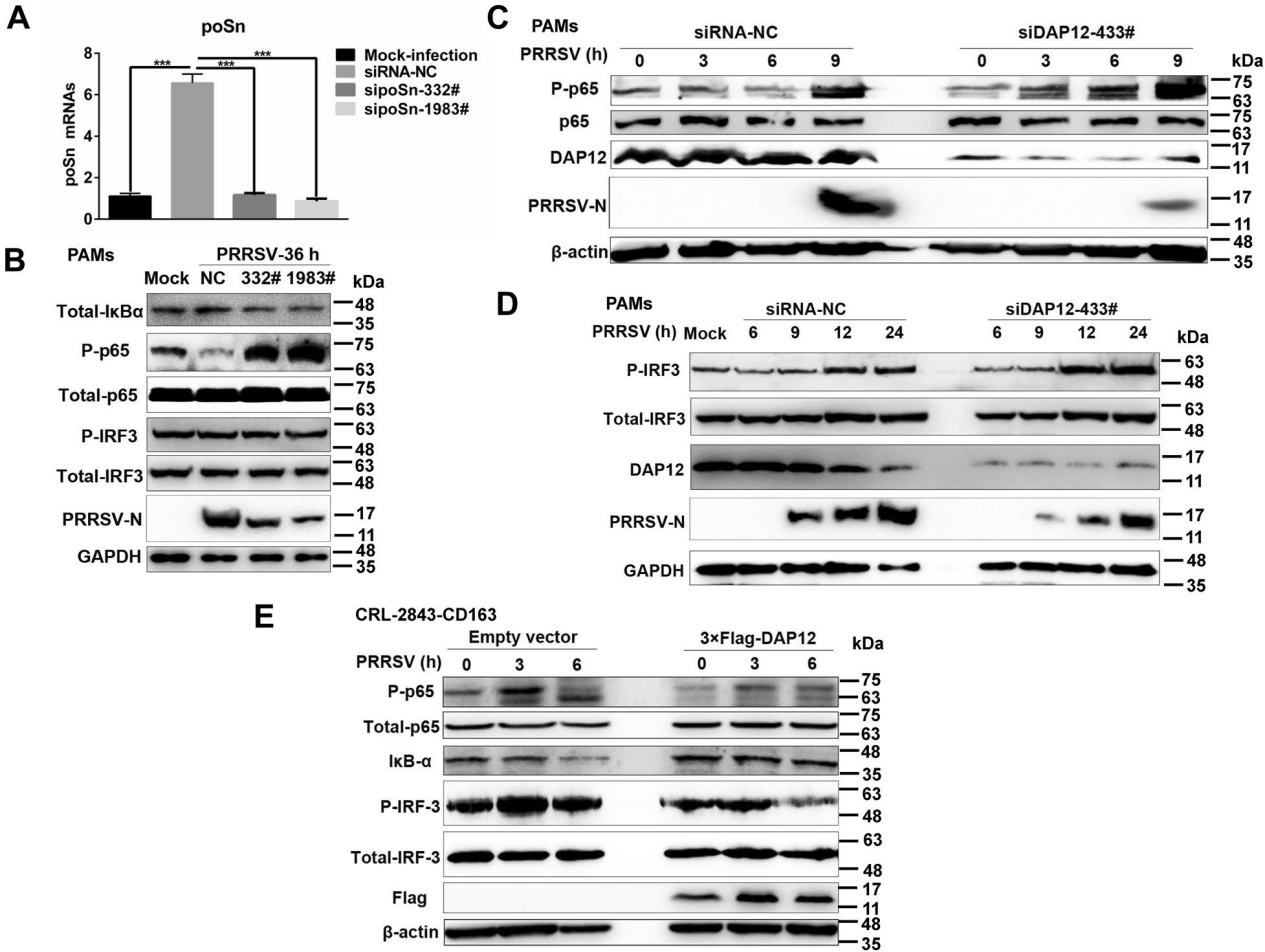
**poSn-DAP12 pathway mediates inhibition of NF-κB in response to PRRSV.A, B** PAMs were pre-incubated with PRRSV (MOI = 1) for 1 h. After washing with serum free medium extensively, the cells were transfected with the indicated sipoSns for 35 h. *poSn* knockdown efficiencies were examined by RT-qPCR (**A**). IB analysis was performed to examine the expression of PRRSV N protein, and the activation of IRF-3 and NF-κB (**B**). **C**, **D***DAP12* knockdown PAMs were infected with PRRSV (MOI = 1) for indicated time periods (0, 3, 6, 9, 12, 24 h). IB was conducted to determine the abundance of PRRSV N protein (**C** ,**D**), and the activation of NF-κB (**C**) and IRF-3 (**D**). **E** DAP12-overexpressed CRL-2843-CD163 cells were infected with PRRSV (MOI = 5) for the indicated time periods (0, 3, 6 h). IB was adopted to detect IκB-α degradation and phosphorylation of IRF-3 and p65. IB panels were representative from three independent experiments. RT-qPCR data were indicated as mean ± SEM. Statistical significances were shown by the Student *t* test: ***p <0.001.

NF-κB and IRF-3 are key transcription factors for type I IFN production [25]. Therefore, we evaluated whether poSn-DAP12 pathway suppressed type I IFN production by impairing NF-κB and IRF-3 activation triggered by PRRSV. We first investigated the effects of *poSn* knockdown on their activation. We pre-incubated PAMs with PRRSV followed by knockdown assays. *poSn* knockdown (Figure 6A) augmented p65 phosphorylation via promoting IκB-α degradation, while the IRF-3 phosphorylation was not influenced (Figure 6B). PRRSV infection was inhibited indicated by the decreased PRRSV N protein expression in *poSn* knockdown PAMs (Figure 6B).

Additionally, we examined the role of DAP12 in PRRSV-triggered NF-κB and IRF-3 activation. We inoculated untreated or *DAP12* knockdown PAMs with PRRSV. *DAP12* knockdown enhanced the phosphorylation of p65 and IRF-3, while suppressing PRRSV infection (Figures 6C and D). On the contrary, we found that DAP12 overexpression in CRL-2843-CD163 cells suppressed p65 phosphorylation by reducing IκB-α degradation, as well as IRF-3 phosphorylation, during PRRSV early infection (Figure 6E).

Collectively, all these results indicate that the poSn-DAP12 pathway participates in inhibiting NF-κB-mediated type I IFN signaling during PRRSV infection.

### poSn-DAP12 participates in antagonism of type I IFN production in response to poly (I:C)

**Figure 7:**
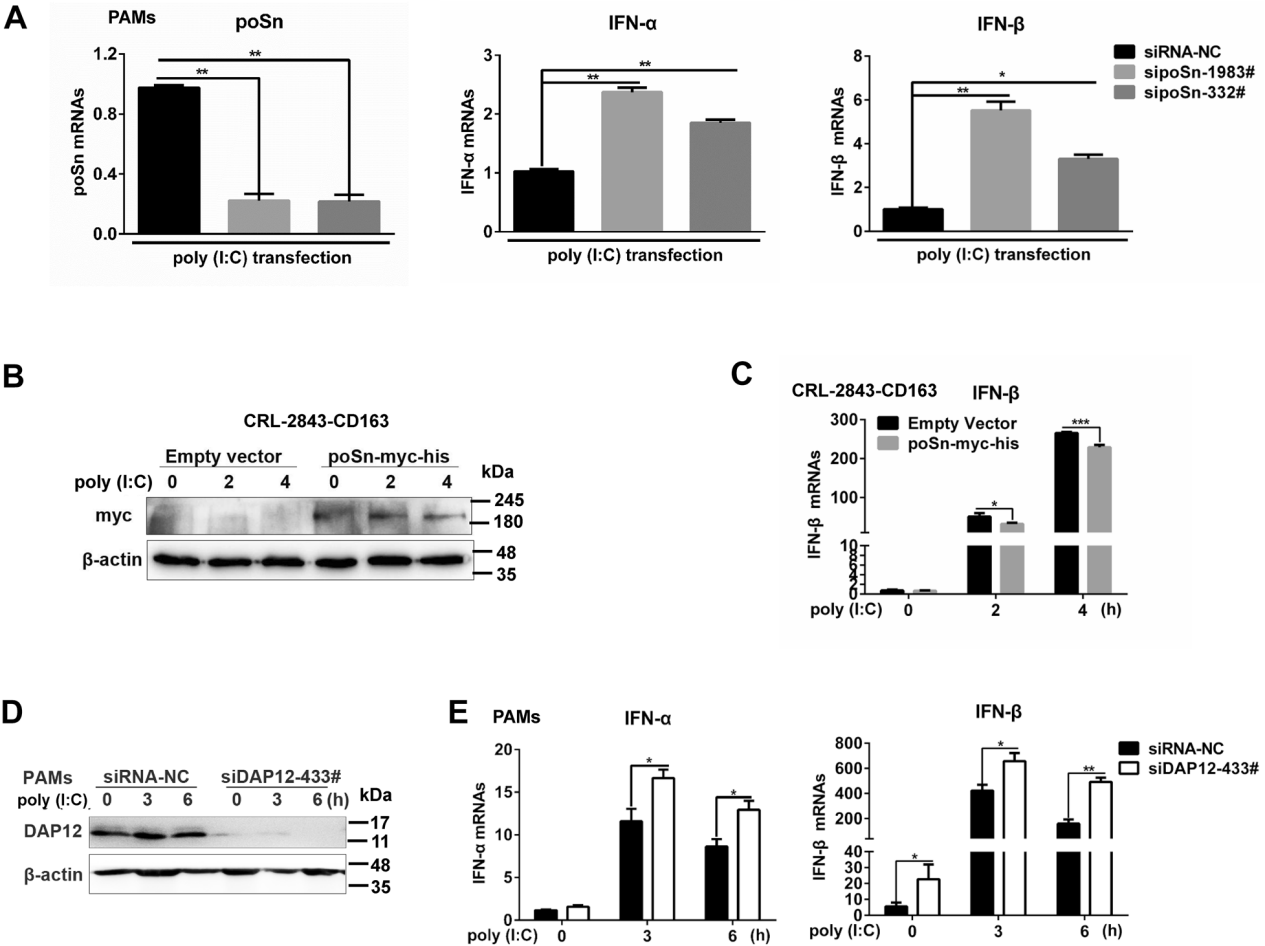
**poSn-DAP12 pathway mediates the inhibition of type I IFN production in response to poly (I:C).A** PAMs were transfected with sipoSn-332# or sipoSn-1983# for 36 h, and then stimulated with 0.25 μg/mL poly (I:C) for 12 h. RT-qPCR analysis was performed to measure the mRNA abundance of poSn and IFN-α/β. **B**, **C** CRL-2843-CD163 cells with poSn overexpression were transfected with 2.5 μg/mL poly (I:C) for the indicated time periods (0, 2, 4 h). poSn expression was determined by IB (**B**). *IFN*-*β* transcription was examined by RT-qPCR (**C**). **D**, **E***DAP12* knockdown PAMs were stimulated with 2 μg/mL poly (I:C) for the indicated time periods (0, 3, 6 h). *DAP12* knockdown was determined by IB (**D**). *IFN*-*α/β* transcription was detected by RT-qPCR (**E**). Quantification data were indicated as mean ± SEM. Statistical significances were shown by Student *t* test: *p < 0.05, **p <0.01, ***p <0.001.

To further probe the IFN-suppressive role of poSn, we examined the effects of *poSn* knockdown on poly (I:C)-stimulated type I IFN production in PAMs. As shown in Figure 7A, knockdown of poSn increased the mRNA abundance of IFN-α/β in response to poly (I:C). On the contrary, poSn overexpression in CRL-2843-CD163 cells attenuated *IFN*-*β* transcription triggered by poly (I:C) (Figures 7B and C). In the following experiments, we investigated the effects of *DAP12* knockdown on type I IFN production triggered by poly (I:C). *DAP12* knockdown promoted *IFN*-*α/β* transcription during poly (I:C)-stimulated periods (Figures 7D and E). In summary, these data suggest that poSn-DAP12 pathway participates in suppressing type I IFN production in response to poly (I:C).

## Discussion

Viral infections usually elicit host innate immune responses, including type I IFN (such as IFN-α/β) production [26, 27]. Various pattern recognition receptors (PRRs) sense the pathogen-associated molecular patterns (PAMPs) to induce activation of IRF-3 or NF-κB [28, 29], which promotes the transcription of type I IFNs and other cytokines [30]. PRRSV has evolved various strategies, such as targeting NF-κB- and/or IRF-3/7-mediated signaling pathways, to antagonize the production of type I IFNs [31–33]. Here, we reveal a novel strategy in which PRRSV exploits poSn to negatively regulate host innate immune responses.

We first explored the biological role of poSn in PRRSV infection, and found that *poSn* knockdown inhibited PRRSV infection by promoting production of type I IFNs in PAMs (Figure 1). Furthermore, we demonstrated that poSn suppressed type I IFN production in response to poly (I:C) (Figure 7), suggesting that poSn-mediated inhibition of IFN responses might be a generic mechanism for host immune regulation. Our study actually uncovered that poSn plays an immunosuppressive role instead of an essential receptor during PRRSV infection.

DAP12 is an immune adaptor involved in modulating innate immune responses [34]. In most cases, DAP12-associated receptors recruit DAP12 and activate the innate immune responses upon recognizing PAMPs [35, 36]. In contrast, we here demonstrated that DAP12 suppressed the virus-triggered type I IFN responses, which was consistent with other reports [37, 38]. *DAP12* knockdown restricted PRRSV infection by increasing type I IFN production (Figures 5A–D), which was similar to the effects of *poSn* knockdown (Figure 1). Since some previous studies indicated that murine Siglec-1 or human Siglec-H interacts with DAP12 to attenuate IFN responses [14, 15, 39, 40], we explored the interaction between poSn and DAP12 during PRRSV infection. We first observed the co-localization of poSn and DAP12 during viral infection (Figure 2A). Subsequently, we confirmed the interaction between poSn and DAP12 by Co-IP (Figure 2B). Moreover, dual-luciferase reporter assays in CRL-2843-CD163 cells indicated that co-expression of poSn and DAP12 inhibited pRIG-I-mediated pig IFN-β promoter activation (Figure 5E). These findings demonstrate that the poSn-DAP12 pathway is involved in dampening type I IFN production, which might be exploited by PRRSV for persistent infection.

In the current study, we further showed that poSn interacted with DAP12, which was dependent on poSn TCD and DAP12 TMD (Figures 2 and 3). We constructed the eukaryotic plasmid poSn without TMD, but failed to express the indicated protein in HEK-293T cells. We hypothesized that poSn TMD was critical for expression and responsible for the interaction between poSn and DAP12. In general, DAP12 D50 is essential for its association with some receptors possessing positively charged residues in their TMDs [35]. We found that there were no positively charged residues in poSn TMD (UniProt entry A7LCJ3), and DAP12 D50 was dispensable for the interaction. Furthermore, we constructed three DAP12 truncations where certain residues within their TMDs were deleted. After a series of Co-IP experiments, we proved that residues 51–57 are critical for the interaction (Figure 4). In another paper, we also found that DAP12 with deletion of residues 51–57 was unable to interact with nonmuscle myosin heavy chain IIA [17]. According to these findings, we speculate that DAP12 residues 51–57 are essential for its interaction with some DAP12-associated receptors, which do not possess any positively charged residues in their TMDs [36].

Since NF-κB or IRF-3-mediated type I IFN production is a classical antiviral response [41, 42], we hypothesized that PRRSV utilized poSn-DAP12 pathway to inhibit their activation, thereby decreasing the production of IFN-α/β. As expected, *poSn* knockdown promoted PRRSV-triggered NF-κB activation by inducing IκB-α degradation (Figures 6A and B), while IRF-3 activation was unaffected. Furthermore, we found that *DAP12* knockdown increased the phosphorylation of p65 and IRF-3 in response to PRRSV (Figures 6C and D), while DAP12 overexpression inhibited their activation (Figure 6E). The poSn-DAP12 pathway indeed contributed to PRRSV infection (Figures 6B–D). The divergence between *poSn* knockdown and *DAP12* knockdown suggested that DAP12 might be involved in various negative regulation pathways mediated by other unidentified receptors. All these findings revealed that the poSn-DAP12 pathway is involved in suppression of PRRSV-triggered NF-κB activation for viral infection. In fact, we have not figured out the underlying mechanism of how poSn-DAP12 pathway influences activation of NF-κB. This work will be our next issue to be resolved.

**Figure 8:**
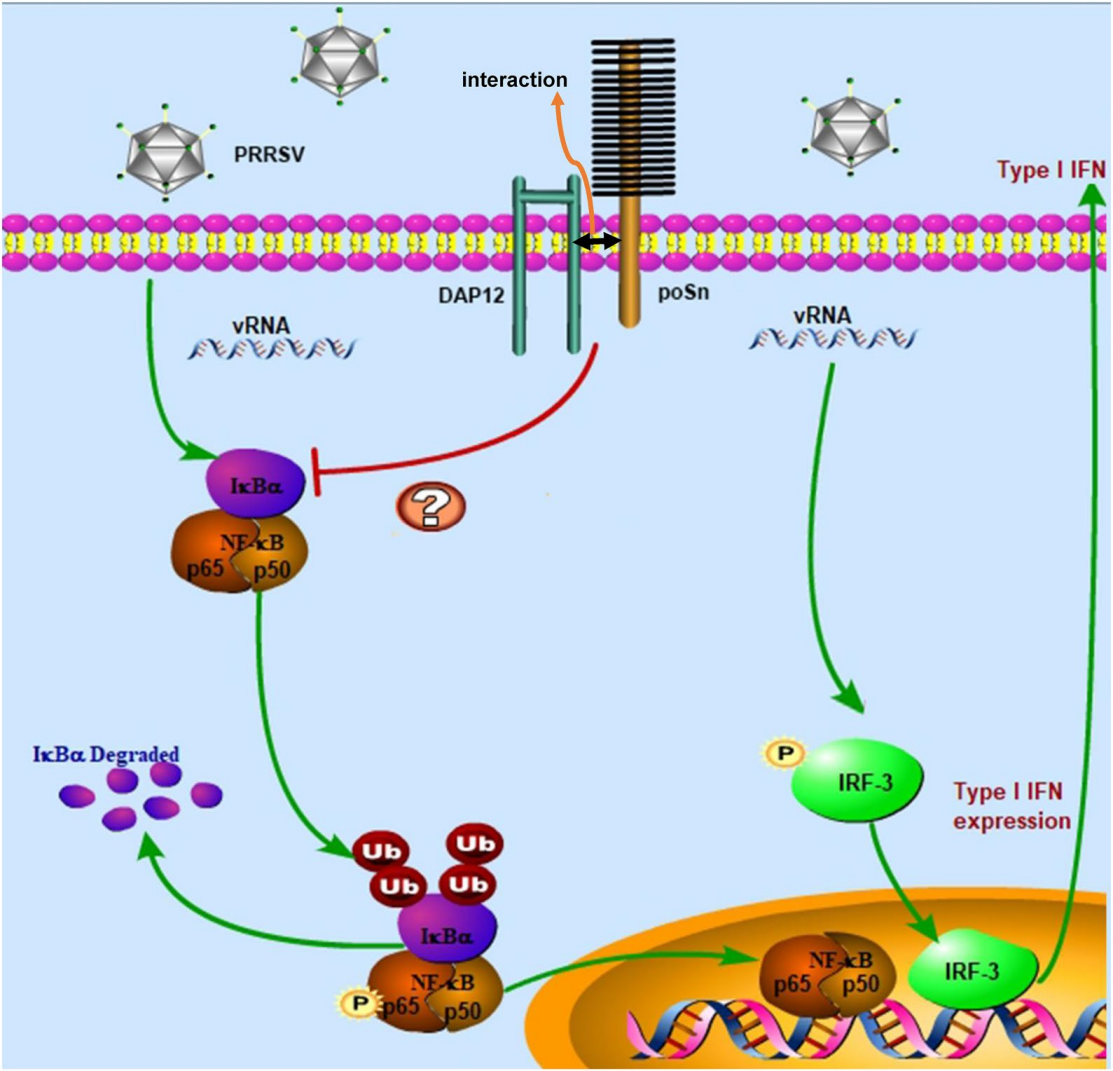
**PRRSV exploits poSn-DAP12 pathway to attenuate type I IFN production for its infection**. poSn associated with DAP12 during PRRSV infection. poSn-DAP12 pathway was exploited by PRRSV to antagonize PRRSV-triggered NF-κB activation, resulting in decreased type I IFN production.

Taken together, we reveal an unappreciated role of poSn in suppressing host innate immune responses during PRRSV infection (Figure 8). poSn interacts with DAP12 through poSn TCD and DAP12 TMD during the PRRSV-post entry process. The poSn-DAP12 pathway targets NF-κB activation to facilitate the viral infection. More importantly, the pathway is involved in antagonizing type I IFN production stimulated by poly (I:C). All these data contribute to the understanding of PRRSV pathogenesis and provide a molecular basis for viral prevention and control.

## Abbreviations

PRRSV: porcine reproductive and respiratory syndrome virus
poSn: porcine sialoadhesin
IFN: type I interferon
DAP12: DNAX-activation protein of 12 kDa
TMD: transmembrane domain
NF-κB: nuclear factor-kappa B
Ig: immunoglobulin
Siglec: sialic acid-binding Ig-like lectin
RIG-I: retinoic acid-inducible gene-I
TBK1: tank binding kinase 1
IRF: interferon regulatory factor
VSV: vesicular stomatitis virus
DMEM: Dulbecco’s modified Eagle’s medium
RPMI-1640: Roswell Park Memorial Institute-1640 medium
FBS: fetal bovine serum
PAMs: pulmonary alveolar macrophages
TCID_50_: 50% tissue culture infective dose
mAb: monoclonal antibody
BSA: bovine serum albumin
RT-qPCR: quantitative real-time PCR
ORF: open reading frame
GAPDH: glyceraldehyde-3-phosphate dehydrogenase
IB: immunoblotting
SDS-PAGE: sodium dodecyl sulfate-polyacrylamide gel electrophoresis
PVDF: polyvinylidene fluoride
PBST: phosphate-buffered saline-Tween 20
RT: room temperature
HRP: horse radish peroxidase
ECL: enhanced chemiluminescence
FCM: flow cytometry
siRNA: small interfering RNA
ECD: extracellular domain
TCD: helical transmembrane plus cytoplasmic domain
IFA: indirect immunofluorescence assay
Co-IP: co-immunoprecipitation
TBST: Tris-buffered saline with 0.5% Tween-20
N: nucleocapsid
ICD: intracellular domain
IκB: NF-κ light polypeptide gene enhancer in B-cells inhibitor
PRRs: pattern recognition receptors
PAMPs: pathogen-associated molecular patterns
ORF: open reading frame
